# Nasal Administration of Cholera Toxin as a Mucosal Adjuvant Damages the Olfactory System in Mice

**DOI:** 10.1371/journal.pone.0139368

**Published:** 2015-09-30

**Authors:** Yoshiko Fukuyama, Kazunari Okada, Masahiro Yamaguchi, Hiroshi Kiyono, Kensaku Mori, Yoshikazu Yuki

**Affiliations:** 1 Division of Mucosal Immunology, Institute of Medical Science, The University of Tokyo, Tokyo, Japan; 2 Department of Physiology, Graduate School of Medicine, The University of Tokyo, Tokyo, Japan; 3 International Research and Development Center for Mucosal Vaccine, The Institute of Medical Science, The University of Tokyo, Tokyo, Japan; Monell Chemical Senses Center, UNITED STATES

## Abstract

Cholera toxin (CT) induces severe diarrhea in humans but acts as an adjuvant to enhance immune responses to vaccines when administered orally. Nasally administered CT also acts as an adjuvant, but CT and CT derivatives, including the B subunit of CT (CTB), are taken up from the olfactory epithelium and transported to the olfactory bulbs and therefore may be toxic to the central nervous system. To assess the toxicity, we investigated whether nasally administered CT or CT derivatives impair the olfactory system. In mice, nasal administration of CT, but not CTB or a non-toxic CT derivative, reduced the expression of olfactory marker protein (OMP) in the olfactory epithelium and olfactory bulbs and impaired odor responses, as determined with behavioral tests and optical imaging. Thus, nasally administered CT, like orally administered CT, is toxic and damages the olfactory system in mice. However, CTB and a non-toxic CT derivative, do not damage the olfactory system. The optical imaging we used here will be useful for assessing the safety of nasal vaccines and adjuvants during their development for human use and CT can be used as a positive control in this test.

## Introduction

The delivery routes and adjuvants used for vaccination are important for the development of protective immune responses against mucosal pathogens. Nasal administration is one of the most effective routes for the induction of antigen-specific protective immunity in both the systemic and mucosal compartments. Because most vaccines are insufficient to induce antigen-specific responses in both the systemic and mucosal immune systems when antigen is administered alone, adjuvant is required to enhance the immune responses. Co-administration of a biologically active mucosal adjuvant, such as cholera toxin (CT) or heat-labile enterotoxin (LT), whose sequence is 80% homologous to that of CT, can overcome the limited response of the mucosal immune system to antigen [1]. In mice and macaques, nasal immunization that includes CT or LT as a mucosal adjuvant induces antigen-specific IgG and secretory IgA (SIgA) and Th2-type cytokine responses [2–5].

CT and LT are potent mucosal adjuvants, but both induce severe diarrhea in humans when administered orally [6–9]. However, they were not known to have any toxicity when administered nasally until an inactivated nasal influenza vaccine containing LT was linked to several cases of Bell’s palsy (facial paralysis) in a clinical study in Switzerland [10]. To avoid the possibility of nasal toxicity with the native toxins, genetically altered nontoxic derivatives of mutant CT (S61F, E112K), mutant LT (R192G), and mutant chimera CTA (E112K)-LTB that have various levels of residual enzymatic activity have been developed [3, 11–13], but the toxicity of nasally administered LT or CT has not yet been fully clarified. The non-toxic derivatives of CT, as well as CT and CTB, reportedly are deposited in the central nervous system (CNS) and accumulate there after nasal administration. Specifically, after the CT or LT molecules bind to GM1 gangliosides, they are transported directly from the nasal mucosa to the olfactory bulbs (OBs), followed by retrograde transport into the olfactory neurons [14–17]. Confirming that CT or CT derivatives do not impair olfactory function will be important for assessing safety after nasal administration of vaccines and adjuvants. Methods for assessing safety in preclinical studies of nasal vaccines and adjuvants will also be needed.

In this study, we used immunohistochemical analyses to examine the pharmacological effects of nasal CT and CT derivatives on the olfactory system. By using optical imaging and an olfactory habituation—dishabituation test in mice, we discovered that nasally administered CT, but not CTB or a non-toxic CT derivative, impaired odor responses in the olfactory system. The optical imaging test may also be useful for assessing the safety of nasal vaccines and adjuvants.

## Materials and Methods

### Mice

Male BALB/c mice (6 to 8 weeks old) were purchased from CLEA (Tokyo, Japan). All experiments were performed in accordance with the Guidelines for Use and Care of Experimental Animals and were approved by the Institutional Animal Care and Use Committee of the University of Tokyo (approval nos. PA09-52 and PA15-44). Mice were maintained at the controlled conditions (room temperature; 22°C, 12:12-h light: dark cycle). Mice had free access to food and water. Mice were euthanized by cervical dislocation under anesthesia with isoflurane.

### Nasal administration

CT was purchased from List Biological Laboratories (Campbell, CA). recombinant CTB, recombinant LTB, and the non-toxic mutant chimera CTA (E112K)-LTB were expressed in *Bacillus brevis* and purified by using immobilized galactose (Pierce, Rockford, IL) in our laboratory [13, 18]. Non-anesthetized mice were nasally administered 30 μg of CT (6 μl of 5 mg/ml, 3 μl per nostril), CTB (10 μl of 3 mg/ml, 5 μl per nostril), LTB (12 μl of 2.5 mg/ml, 6 μl per nostril) or CTA (E112K)-LTB (12 μl of 2.5 mg/ml, 6 μl per nostril) by using a pipette to deliver the fluid dropwise into each nostril. At 1 day (24 h) or 3 days (72 h) after nasal administration, mice were used in experiments.

### Pathology and immunohistochemistry

Mice were transcardially perfused with phosphate-buffered saline (PBS) followed by 4% paraformaldehyde. For sectioning the OBs, the dissected brains were fixed in 4% paraformaldehyde in PBS overnight at 4°C, washed in PBS, and equilibrated in 30% sucrose in PBS before they were mounted in OCT compound (Sakura Finetek, Tokyo, Japan). For sectioning the olfactory epithelium (OE), the nasal tissue was decalcified with 0.5 M EDTA in 30% sucrose in PBS at 60°C for 2 days. Frozen sections (OE, 20 μm; OB, 12 μm) were stained with 5 μg/ml of an anti-CTA rabbit antibody (Ab), anti-CTB rabbit Ab, or anti-LTB rabbit Ab (protein A purified, our laboratory) followed by Alexa 488–conjugated rabbit IgG (Invitrogen, Carlsbad, CA); an anti-olfactory marker protein (OMP) Ab (1:2000, Wako, Osaka, Japan) followed by Alexa 546–conjugated goat IgG (Invitrogen); or an anti-s-100β monoclonal Ab (1:200, Abcam, Cambridge, UK) followed by Alexa 647–conjugated mouse IgG (Invitrogen). Frozen OE sections were stained with hematoxylin and eosin (H&E) to detect histological lesions.

### Quantification of OMP expression levels

The numbers of OMP-positive cells located within the OE were counted. OMP expression levels in the OB were quantified by using Adobe Photoshop Elements 10 (Adobe Systems Software) as described previously [19]. By using the Histogram tool, the total number of pixels and the number of pixels positive for OMP signals were counted, and the signal intensity of OMP was calculated as a percentage of the total area in the glomeruli of the OBs that was positive for immunohistochemical signals.

### Image acquisition and analysis

Images of entire OB sections were obtained with fluorescent microscopy (DM6000B; Leica, Wetzlar, Germany) under a 10× objective lens with MetaMorph (Molecular Devices). Images of OE sections and glomeruli in OB sections were obtained with confocal laser scanning microscopy (TCS SP5; Leica) under an oil-immersion 63× objective lens with LAS AF software (Leica). The pictures of glomeruli in OB sections are shown in black and white to visually improve signal sharpness without manipulating signal intensity or contrast [20]. H&E sections were visualized and the OE thickness was analyzed under a light microscope (BX53; Olympus, Tokyo, Japan) by using cellSens software (Olympus).

### Olfactory habituation–dishabituation test

The olfactory habituation—dishabituation test was performed as described previously [21]. Mice were nasally administered 30 μg of CT, CTB, LTB or CTA (E112K)-LTB and then habituated to the experimental cage. Next, a sheet of filter paper (2 × 2 cm) with 20 μl of mineral oil (Sigma—Aldrich, St. Louis, MO) was placed on the bottom of the cage for 3 min. This procedure was performed three times with 15-min intervals. In the fourth trial, filter paper with 20 μl of cheese odor (Hasegawa Company, Tokyo, Japan) was presented for 3 min; the presentation was repeated in the fifth and sixth trials, with 15 min intervals between trials. In the seventh, eighth, and ninth trials, shrimp odor (Hasegawa Company) was presented. A mouse was judged to be investigating the stimulus when its nose came within 1 mm of the filter paper. Investigation time during a 3-min test period was measured. To avoid confounding of data owing to learning, mice were used only once.

### Optical imaging of intrinsic signals

Detailed procedures for optical imaging have been described previously [22–25]. Mice were nasally administered 30 μg of CT or CTB and then anesthetized at 24 h or 72 h with medetomidine (0.5 mg/kg), ketamine (22.5 mg/kg), and sodium pentothal (25 mg/kg). The skull overlying the dorsal surfaces of both OBs was removed. Agarose gel was mounted on both OBs and covered with a cover glass. Images of reflected light from the dorsal surface of the OBs were collected by using a CCD camera (CS8310; Tokyo Electronic Industry, Tokyo, Japan) and digitized with an IBM/PC-compatible computer equipped with a video frame-grabber board (Pulsar; Matrox Graphics, Quebec, Canada). A 4.2 × 3.1 mm region was imaged at a spatial resolution of 320 × 240 pixels. Intrinsic signals were imaged with 705-nm-wavelength light, and the focusing depth was adjusted to 50–150 μm below the dorsal surface of the OB to avoid excess background noise. For each recording trial, data were collected for 8 s, with a frame length of 0.5 s (16 frames per trial). Odorant stimulation was applied from the beginning of the fourth to the end of the sixteenth frame. Two aliphatic acid odorants, propionic acid (3COOH) and valeric acid (5COOH) (Sigma—Aldrich), were used, and each stimulation was performed by placing an odorant-containing test tube within 10 mm of the mouse’s nostrils. Images were analyzed with IDL software (Research Systems, Boulder, CO) as described previously [22, 23, 25]. To avoid confounding of data owing to learning, mice were used only once.

### Statistical analysis

All data are presented as means ± standard deviations (SD). The results for the quantification of OMP expression levels and OE thickness were compared by using Student’s *t*-test, and the data from the habituation—dishabituation test were compared by using one-way ANOVA followed by the Dunnett test. Comparisons yielding *P* values less than 0.05 were considered significant.

## Results

### Nasal administration of CT, but not CTB or a non-toxic CT derivative, impairs olfactory sensory neurons in mice

**Fig 1:**
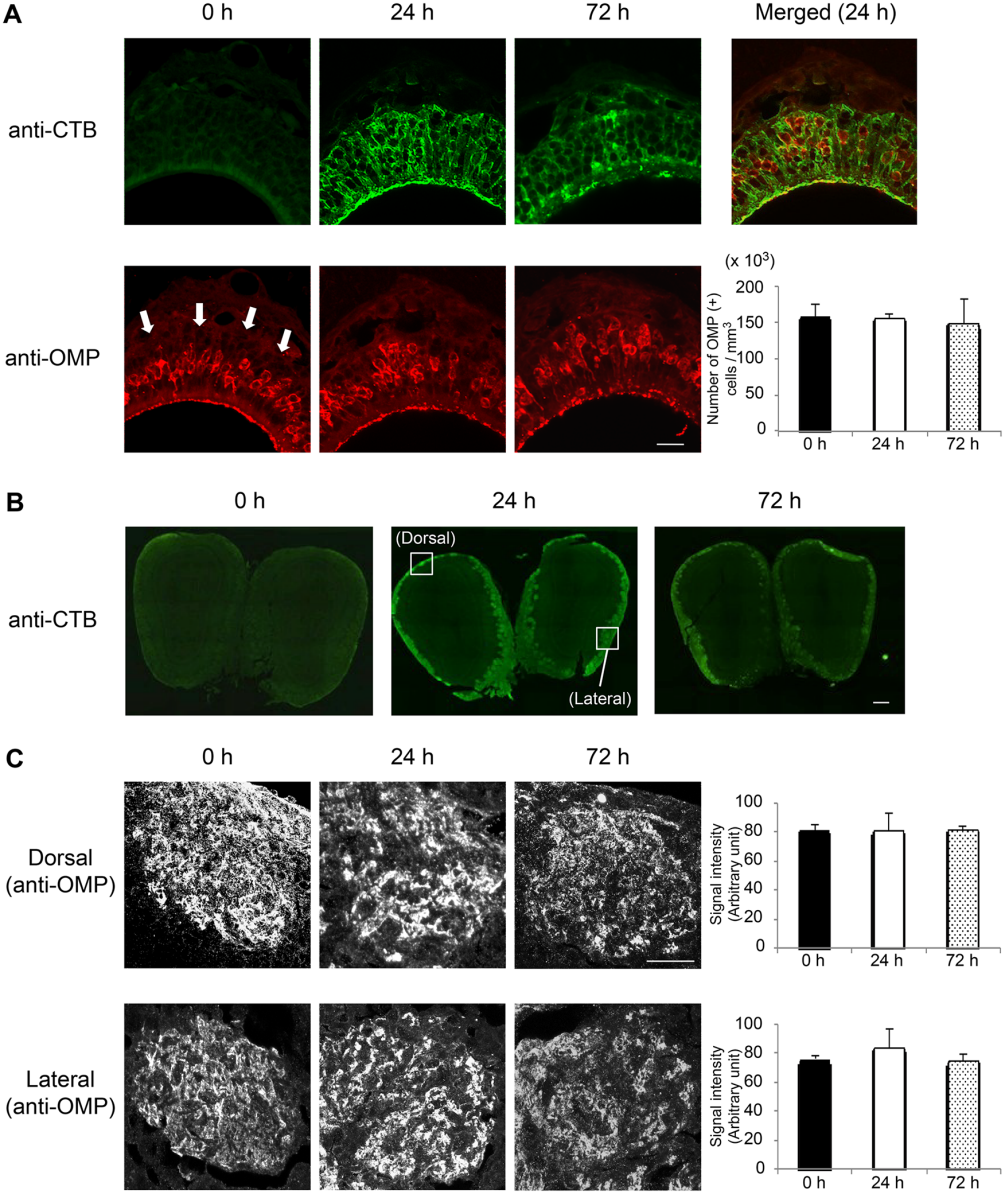
Immunohistochemical staining of the OE and OBs after nasal administrationof 30 μg CTB. OE and OBs were obtained at 0 (untreated), 24, or 72 h after nasal administration of 30 μg CTB. (**A**) Frozen sections of OE were stained with an anti-CTB Ab (green) or anti-OMP Ab (red). White arrows indicate the olfactory nerve layer. The number of OMP-positive cells (mean ± 1 SD; *n* = 3 mice) in each experimental group is shown at the right. (**B**) Frozen sections of OBs were stained with an anti-CTB Ab. (**C**) Frozen sections of OBs were stained with an anti-OMP Ab (converted to black and white images). Glomeruli at the dorsal and lateral surfaces of the OBs (indicated by the boxes in panel B) are shown. The signal intensity (mean ± 1 SD; *n* = 3 mice) in each experimental group is shown. Data are representative of three independent experiments. Scale bars: (A, C) 20 μm, (B) 200 μm.

**Fig 2:**
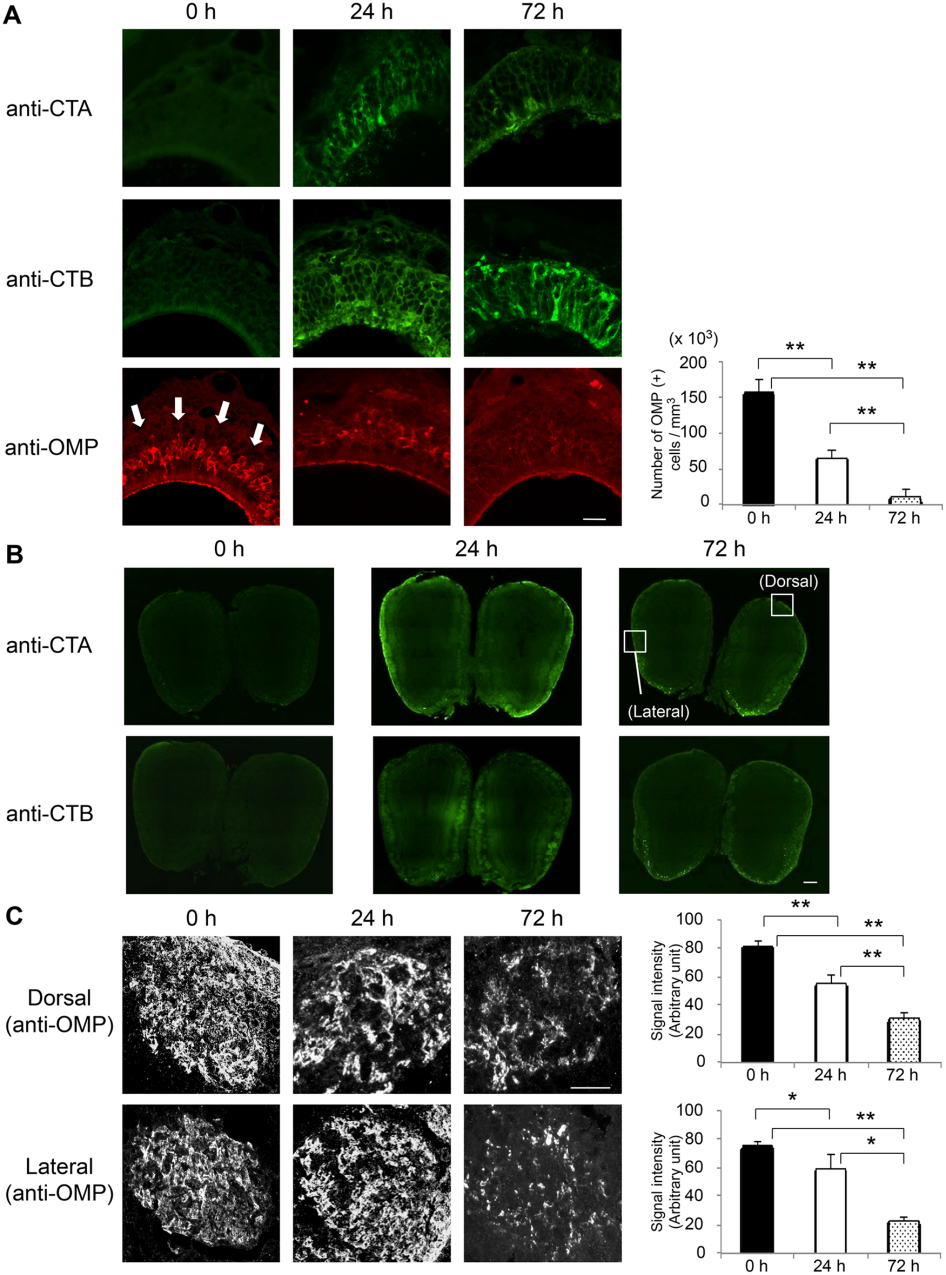
Immunohistochemical staining of the OE and OBs after nasal administration of 30 μg CT. OE and OBs were obtained at 0 (untreated), 24, or 72 h after nasal administration of 30 μg CT. (**A**) Frozen sections of OE were stained with an anti-CTA Ab or anti-CTB Ab (green) or an anti-OMP Ab (red). White arrows indicate the olfactory nerve layer. The number of OMP-positive cells (mean ± 1 SD; *n* = 3 mice) in each experimental group is shown at the right. (**B**) Frozen sections of OBs were stained with an anti-CTA Ab or anti-CTB Ab. (**C**) Frozen sections of OBs were stained with an anti-OMP Ab (converted to black and white images). Glomeruli at the dorsal and lateral surfaces of OBs (indicated by the boxes in panel B) are shown. The signal intensity (mean ± 1 SD; *n* = 3 mice) in each experimental group is shown at the right. Data are representative of three independent experiments. *P < 0.05, **P < 0.01 (Student’s *t*-test). Scale bars: (A, C) 20 μm, (B) 200 μm.

Using immunohistochemical analysis, we confirmed that nasally administered CTB and CT were taken up from the olfactory and respiratory epithelia and deposited in the OBs 24 h after nasal administration (Fig 1A, 1B and Fig 2A, 2B). We next investigated whether the presence of CTB or CT in the OBs impaired olfactory sensory neurons in mice. OMP expression did not change in the OE or in the glomeruli at the dorsal and lateral surfaces of OBs over the 72-h period after nasal administration of 30 μg CTB, although CTB was transported from the OE to the OBs (Fig 1A and 1C). However, OMP expression in the OE gradually disappeared beginning 24 h after nasal administration of 30 μg CT and had completely disappeared by 72 h (Fig 2A). Furthermore, OMP expression disappeared from glomeruli at the dorsal and lateral surfaces of OBs 72 h after nasal administration of 30 μg CT (Fig 2C). H&E staining of the sections showed that OE thickness was significantly lower at 72 h after 30 μg CT administration than in the untreated group or at 72 h after administration of 30 μg CTB (S1D Fig). This result indicates that nasal administration of 30 μg CT induced epithelial damage along virtually the entire lining of the OE. These results suggest that nasal administration of 30 μg CT but not CTB impaired the OE at 72 h and the OBs at 72 h. We hypothesized that CT damages neurons due to the toxicity of CTA. Supporting this, OMP expression was unchanged in the OE or glomeruli at 72 h after nasal administration of 30 μg of a non-toxic mutant chimera, which combines the A subunit of a nontoxic mutant derivative of CT and the B subunit of LT [CTA (E112K)-LTB] [13], although the chimeric adjuvant was transported from the OE to the OBs (S2A and S2C Fig). Like CTB, LTB alone was transported from the OE to the OBs (S3A and S3C Fig). These results indicate that the toxic CTA subunit was responsible for the induced neuronal cell damage.

### Nasal administration of CT, but not CTB or a non-toxic CT derivative, impairs behavioral odor responses

**Fig 3:**
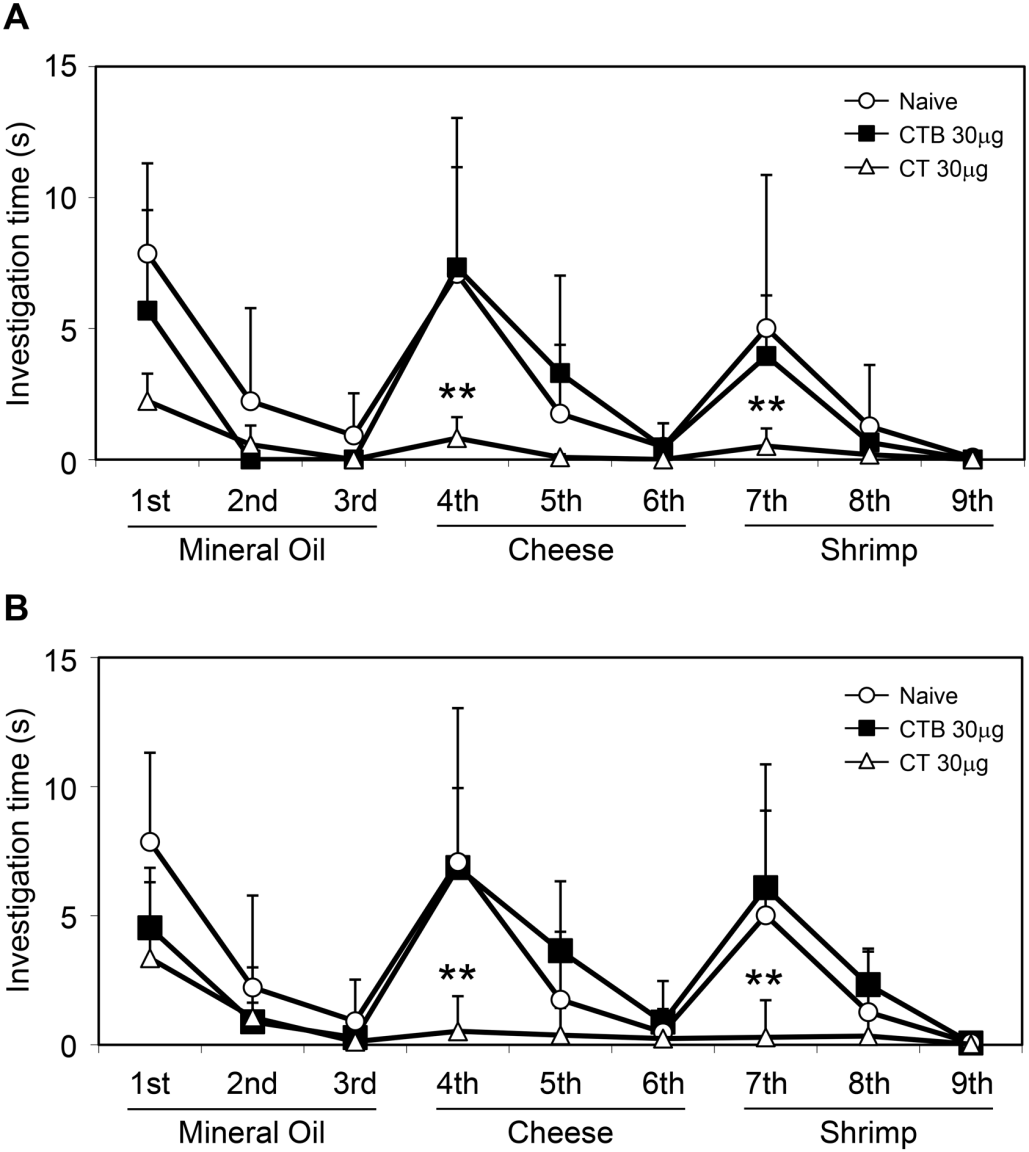
Habituation–dishabituation test after nasal administration of 30 μg CTB or CT. One day (24 h, **A**) or three days (72 h, **B**) after nasal administration of the indicated compounds, the habituation—dishabituation test was performed with the indicated odors. Naïve (untreated) mice were used as controls. For each group, the time spent in investigation (mean ± 1 SD; *n* = 10 mice) in each experimental group during each 3-min period (with 15-min intervals) is shown. **P < 0.01 (one-way ANOVA followed by the Dunnett test) compared with value for naïve mice or mice after nasal administration of CTB.

Next, we investigated whether nasal CT or CTB administration impairs behavioral responses of mice to odors. Mice were habituated to odorless mineral oil in three sessions of 3 min each, with 15-min intervals between sessions, and then exposed to a new odorant, cheese. When naïve mice were exposed to the new odorant, the investigation time increased dramatically (Fig 3A and 3B), indicating that they had detected the new odor. Furthermore, when the naïve mice were exposed to a third odorant, shrimp, after they had habituated to the cheese odor, they were once again able to identify the change, and their investigation time again increased (Fig 3A and 3B). There was no significant difference in the investigation time between naïve mice that did not receive any form of CT and those treated intranasally with 30 μg CTB (Fig 3A and 3B) or 30 μg LTB or 30 μg CTA (E112K)-LTB (S4A and S4B Fig). In contrast, mice given 30 μg CT nasally showed a significantly lower investigation time in response to a new odorant than did naïve mice or mice that received 30 μg CTB nasally when the test was performed at 24 h (Fig 3A) or 72 h (Fig 3B) after the administration of CT or CTB. In addition, it was noted that the responses to trials 4 and 7 (odors) were significantly lower than the response to trial 1 (odorless mineral oil) in the mice given CT. The impairment of behavioral responses to odorants was not due to sickness or diarrhea after nasal administration of 30 μg CT, because there was no significant difference in the behavior of naïve mice and those given CT after exposure to mineral oil, which is odorless (Fig 3A and 3B). These results suggest that nasal administration of CT impairs behavioral responses to odorants, a result that is consistent with the results of the immunohistochemical analysis of OMP staining.

### No odor responses were detected in mice nasally administered CT

**Fig 4:**
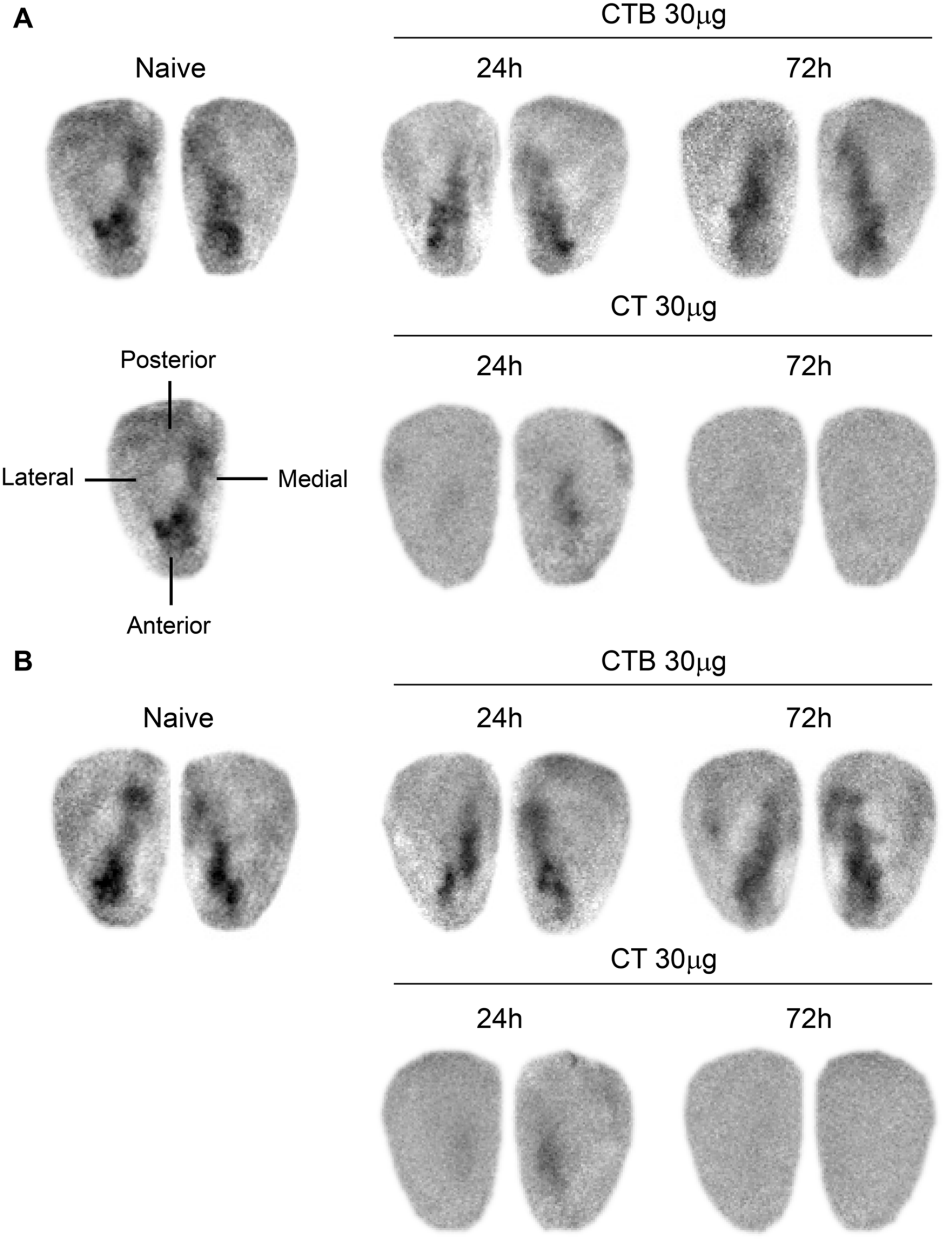
Optical imaging of intrinsic signals in response to odorant stimulation recorded from the dorsal surface of the OB. Spatial distribution of the response to stimulation with propionic acid (**A**) and valeric acid (**B**) recorded from the right and left OBs in naïve mice, or 24 or 72 h after nasal administration of 30 μg of CTB or CT. Data are representative of three independent experiments (*n* = 3 mice).

To confirm the decrease in odorant detection visually, we analyzed odor-activated glomerular maps by optical imaging of intrinsic signals. The aliphatic acids propionic acid (3COOH) and valeric acid (5COOH) activated glomeruli in the anteromedial region of the imaged area, and the response extended to the posterior part of the region in the dorsal surface of the OBs of naïve mice (Fig 4A and 4B). These glomerular responses were also observed at 24 h and 72 h after mice were nasally administered 30 μg CTB (Fig 4A and 4B). However these responses had decreased at 24 h in the mice administered 30 μg CT, and no glomerular responses were detected 72 h after the administration of CT (Fig 4A and 4B). Therefore, nasal administration of 30 μg CT impairs odor detection by damaging the OE and OBs.

## Discussion

We have assessed olfactory function during vaccine and adjuvant development by using an olfactory habituation—dishabituation test and optical imaging in mice. Using these methods, along with CT and CT derivatives as model vaccines and adjuvants, we have demonstrated that nasal administration of CT, but not CTB or a nontoxic CT derivative, damages olfactory sensory neurons (OSNs) and impairs olfactory function.

When CT and CT derivatives are administered nasally, there are two possible routes for the transfer of CT and CTB from the OE to the OB: the extracellular pathway and the intracellular pathway. In the extracellular pathway, nasally administered drugs enter channels created by olfactory ensheathing cells that surround the olfactory nerves, from where they can access the cerebrospinal fluid and OBs [26]. Intracellular transport occurs within the axons of the OSNs, which are located within the OE of the nasal cavity and project their axons into glomerular structures of the OBs [26–31]. Influenza virus (e.g., H5N1 virus) also enters the CNS via the axonal route, moving along the olfactory and trigeminal nerves from the OE to the OBs in mice [32–34] and ferrets [35–37]. Ferrets inoculated with the H5N1 virus showed severe necrosis of OE cells 5 days after inoculation [36]. Therefore, it is important to test whether a nasal drug-delivery system impairs the olfactory nerve system, as the influenza virus does.

Nasally administered CT, but not CTB or CTA (E112K)-LTB, down-regulated the OMP expression level in OSNs (Fig 2A and 2C, S2A and S2C Fig). OSNs have been reported to undergo necrotic cell death when toxic chemicals such as ZnSO_4_ are administered directly to the nasal cavity. Indeed, zinc rapidly induces selective necrosis of olfactory cells because it enters the cells and degrades various cellular components [38]. Divalent cations, including Zn^2+^ and Cd^2+^, are reported to elevate the intracellular Ca^2+^ concentration and induce necrotic cell death [39–41]. In contrast, CTB binds to a GM1 ganglioside on the cell membrane, thus enabling translocation of the toxic A subunit across the membrane into the cell and resulting in intracellular accumulation of cAMP activity via ADP-ribosylation of the stimulatory G protein of adenylyl cyclase, G_s_, and cell intoxication with subsequent inflammatory responses in the nasal tract [15, 42]. The decrease in OMP expression could reflect damage of OE containing neuronal cells and supporting cells (S1C and S1D Fig). In future studies, it will be worth testing whether CT specifically induces OSN cell death as one of the mechanisms of OE damage.

In the mammalian olfactory system, OSNs are located within the OE and project their axons into the glomerular structures of the OBs [26–31]. As many as 1,000 functional odorant receptor genes are expressed in the OE, enabling the detection and discrimination of diverse odorants [43]. In mammalian OBs, many odorants preferentially evoke activity in glomeruli clustered in spatial domains, and these domains are associated with odorants of a particular chemical class, given that the axons of OSNs expressing the same odorant receptor converge on a specific set of glomeruli in the OBs [25, 44, 45]. Therefore, the physical arrangement of glomeruli produces an odorant receptor map in the glomerular layer of the OB [25, 46, 47]. The spatial pattern of the odorant-evoked glomerular activity in mammalian OBs has been analyzed with optical imaging of intrinsic signals [25], and the activity of multiple odorants has been optically mapped [22, 23, 48]. We used this optical imaging technique to evaluate visually whether nasal administration of CT or CTB impairs odor responses of mice. In this study, odor responses were not induced in mice after nasal administration of 30 μg CT, but responses were maintained after administration of CTB (Fig 4). These results are consistent with another study in which odor responses were not visible by optical imaging in mice given a high dose of cadmium (20 μg) nasally, because cadmium exposure damages the connectivity between the OE and OBs [49]. We suggest that when OSNs are impaired by nasal drug administration, they cannot project odor signals to glomeruli in the OBs, resulting in OBs that cannot detect odors. Therefore the optical imaging test may be useful for assessing the safety of novel nasal vaccines and adjuvants.

To examine functional odor responses, we used an olfactory habituation—dishabituation test to assess whether the animals could detect and differentiate novel odors [50, 51]. Habituation is defined by a progressive decrease in olfactory investigation towards a repeated presentation of the same odor stimulus, whereas dishabituation is defined by a reinstatement of investigation when a novel odor is presented. In the current study, after we confirmed that mice had normal investigation and habituation behaviors to an odorless substance (mineral oil), we tested them with odorous materials. When mice given 30 μg of CTB, LTB, or CTA (E112K)-LTB were exposed to a new odorant in the fourth trial, they detected the new odorant, and the investigation time increased (dishabituation; Fig 3 and S4 Fig). In contrast, mice treated with 30 μg of CT showed significantly less investigation time than that of naïve mice or those treated with CTB (Fig 3), indicating that they have impaired odor detection. After habituation to this new odor over three trials, the control mice, such as those treated with CTB, were able to identify a second new odorant in the seventh trial. In contrast, mice treated with 30 μg of CT again showed significantly less investigation of the second new odorant. Although it is currently difficult to determine whether this abnormality includes impaired discrimination or memory [52] of odors in addition to impaired odor detection, these impairments in the olfactory habituation—dishabituation test are consistent with the results of the optical imaging test. CT or CTB administered nasally is not transported to brain in mice [14, 17], so we did not expect any effect of treatment on the brain, including the olfactory cortex. Therefore, we considered that mice administered CT nasally have normal olfactory function in the higher cortices but damaged olfaction in the periphery, from the OE to the OB.

In summary, we have shown that nasal administration of CT results in the transport of the protein to the OBs and impairment of the olfactory nerve system, whereas nontoxic CT derivatives are transported but do not impair olfaction. Because of the adverse CNS effects of CT administered nasally, it may serve as an effective positive control when assessing the safety of nasal vaccines and adjuvants in development for human use.

## Supporting Information

S1 Fig H&E staining of the OE after nasal administration of 30 μg CTB or CT. OE were obtained at 0 h (untreated; **A**) or 72 h after nasal administration of 30 μg CTB (**B**) or CT (**C**). Sections were stained with H&E and visualized under a light microscope. (**D**) OE thickness before and 72 h after nasal administration of 30 μg CTB or CT. Data are representative of three independent experiments (*n* = 3 mice). **P < 0.01 (Student’s *t*-test). Scale bars: 50 μm.
(TIF)

S2 Fig Immunohistochemical staining of the OE and OBs after nasal administration of 30 μg CTA (E112K)-LTB. OE and OBs were obtained at 0 (untreated), 24, or 72 h after nasal administration of 30 μg CTA (E112K)-LTB. (**A**) Frozen sections of OE were stained with an anti-CTA Ab or anti-LTB Ab (green) or an anti-OMP Ab (red). White arrows indicate the olfactory nerve layer. The number of OMP-positive cells (mean ± 1 SD; *n* = 3 mice) in each experimental group is shown at the right. (**B**) Frozen sections of OBs were stained with an anti-CTA Ab or anti-LTB Ab. (**C**) Frozen sections of OBs were stained with an anti-OMP Ab (converted to black and white images). Glomeruli at the dorsal and lateral surfaces of OBs (indicated by the boxes in panel B) are shown. The signal intensity (mean ± 1 SD; *n* = 3 mice) in each experimental group is shown. Data are representative of three independent experiments. Scale bars: (A, C) 20 μm, (B) 200 μm.
(TIF)

S3 Fig Immunohistochemical staining of the OE and OBs after nasal administration of 30 μg LTB. OE and OBs were obtained at 0 (untreated), 24, or 72 h after nasal administration of 30 μg LTB. (**A**) Frozen sections of OE were stained with an anti-LTB Ab (green) or anti-OMP Ab (red). White arrows indicate the olfactory nerve layer. The number of OMP-positive cells (mean ± 1 SD; *n* = 3 mice) in each experimental group is shown at the right. (**B**) Frozen sections of OBs were stained with an anti-LTB Ab. (**C**) Frozen sections of OBs were stained with anti-OMP Ab (converted to black and white images). Glomeruli at the dorsal and lateral surfaces of OBs (indicated by the boxes in panel B) are shown. The signal intensity (mean ± 1 SD; *n* = 3 mice) in each experimental group is shown. Data are representative of three independent experiments. Scale bars: (A, C) 20 μm, (B) 200 μm.
(TIF)

S4 Fig Habituation–dishabituation test. Mice were nasally administered 30 μg LTB or CTA (E112K)-LTB. One day (24 h, **A**) or 3 days (72 h, **B**) after administration of the indicated drugs, the habituation—dishabituation test was performed with the indicated odors. Naïve (untreated) mice were used as controls. For each group, the time spent in investigation (mean ± 1 SD; *n* = 10 mice) in each experimental group during each 3-min period (with 15-min intervals) is shown.
(TIF)
