# Supplementary figures and images for: Nasal Administration of Cholera Toxin as a Mucosal Adjuvant Damages the Olfactory System in Mice
Source: PLoS One. 2015 Sep 30;10(9):e0139368. doi: 10.1371/journal.pone.0139368 (PMC4589288; doi:10.1371/journal.pone.0139368)

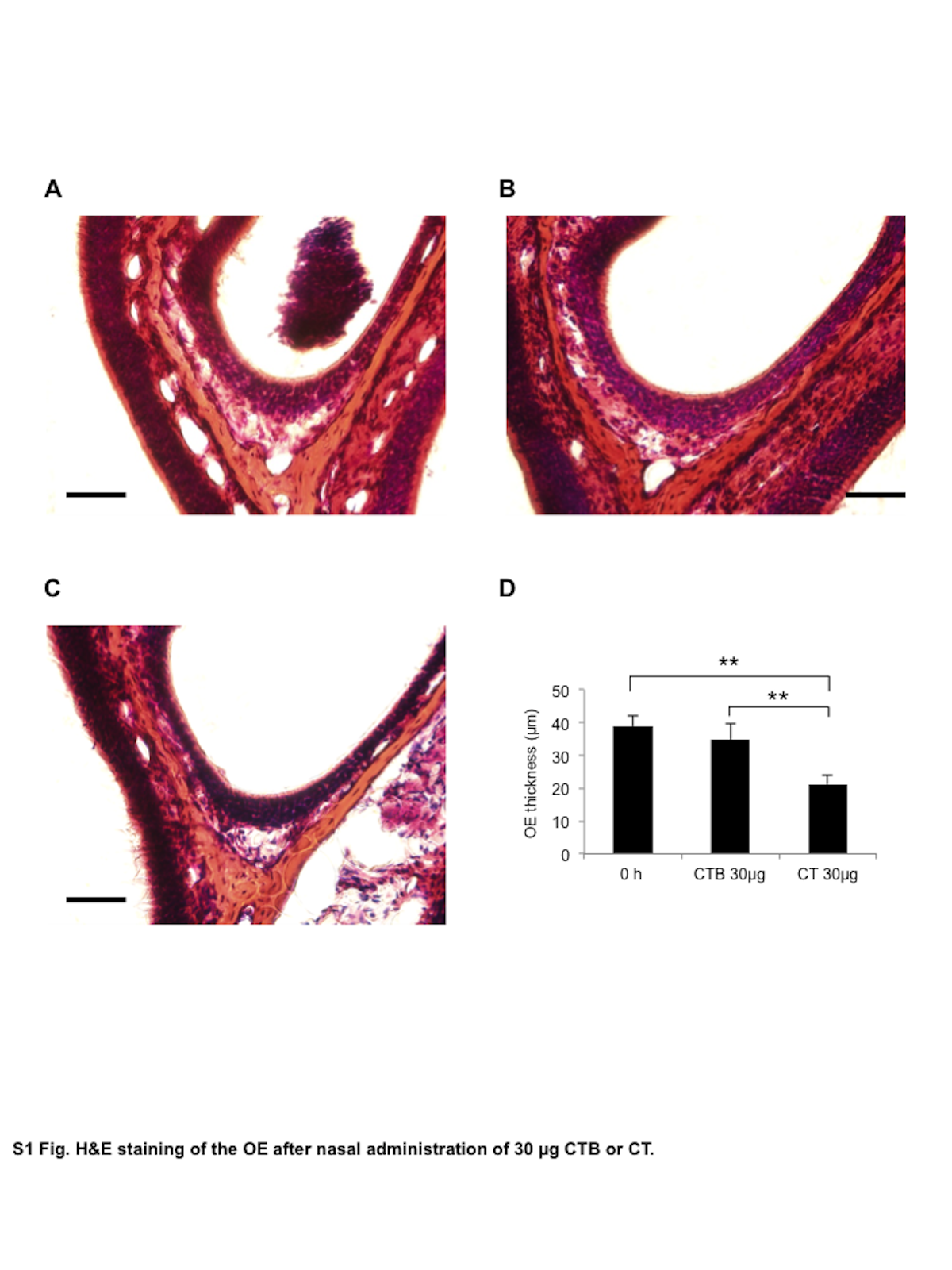

Supplement: S1 Fig — OE were obtained at 0 h (untreated; A) or 72 h after nasal administration of 30 μg CTB (B) or CT (C). Sections were stained with H&E and visualized under a light microscope. (D) OE thickness before and 72 h after nasal administration of 30 μg CTB or CT. Data are representative of three independent experiments (n = 3 mice). **P < 0.01 (Student’s t-test). Scale bars: 50 μm. (TIF) [file pone.0139368.s001.tif]

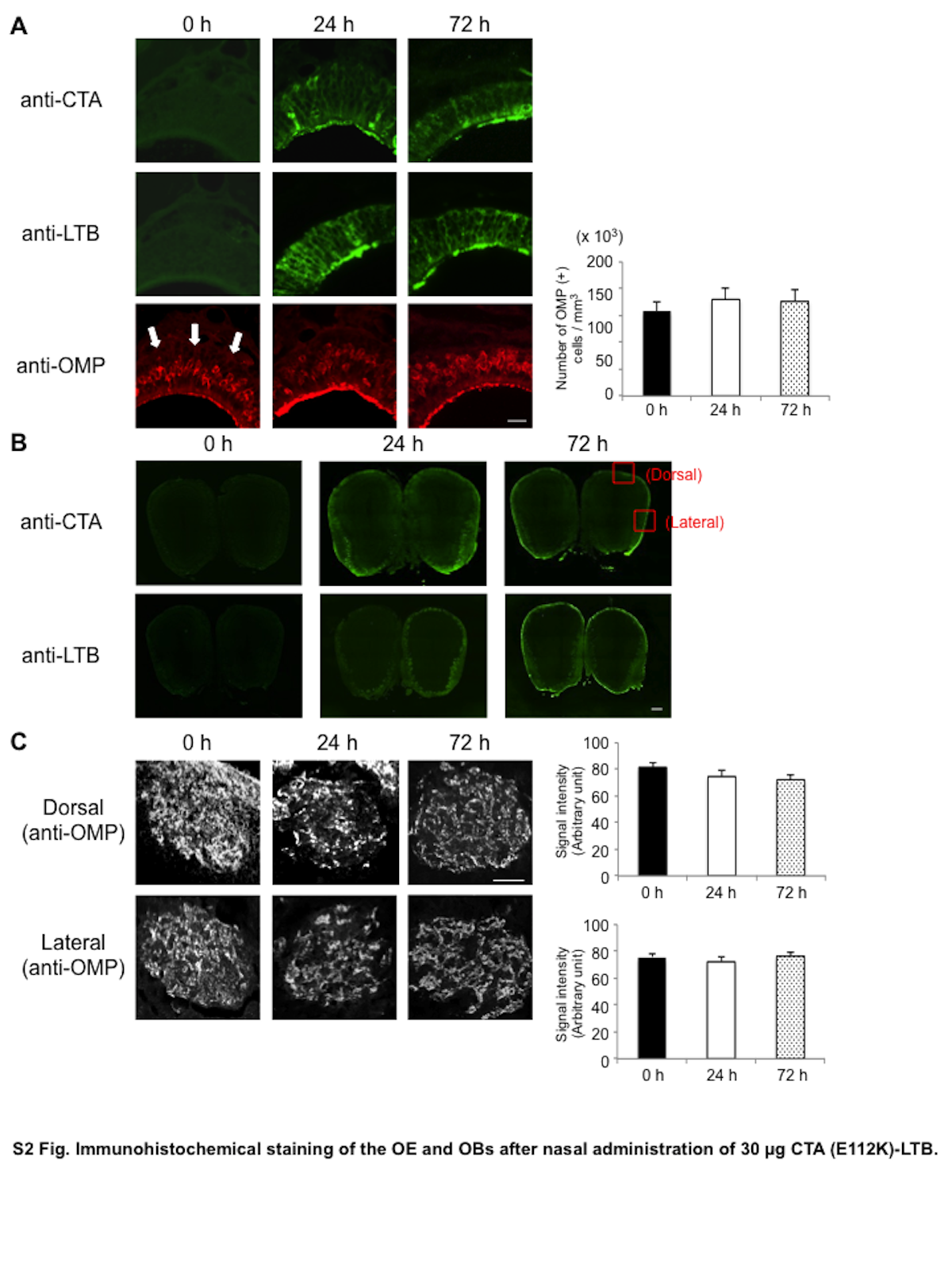

Supplement: S2 Fig — OE and OBs were obtained at 0 (untreated), 24, or 72 h after nasal administration of 30 μg CTA (E112K)-LTB. (A) Frozen sections of OE were stained with an anti-CTA Ab or anti-LTB Ab (green) or an anti-OMP Ab (red). White arrows indicate the olfactory nerve layer. The number of OMP-positive cells (mean ± 1 SD; n = 3 mice) in each experimental group is shown at the right. (B) Frozen sections of OBs were stained with an anti-CTA Ab or anti-LTB Ab. (C) Frozen sections of OBs were stained with an anti-OMP Ab (converted to black and white images). Glomeruli at the dorsal and lateral surfaces of OBs (indicated by the boxes in panel B) are shown. The signal intensity (mean ± 1 SD; n = 3 mice) in each experimental group is shown. Data are representative of three independent experiments. Scale bars: (A, C) 20 μm, (B) 200 μm. (TIF) [file pone.0139368.s002.tif]

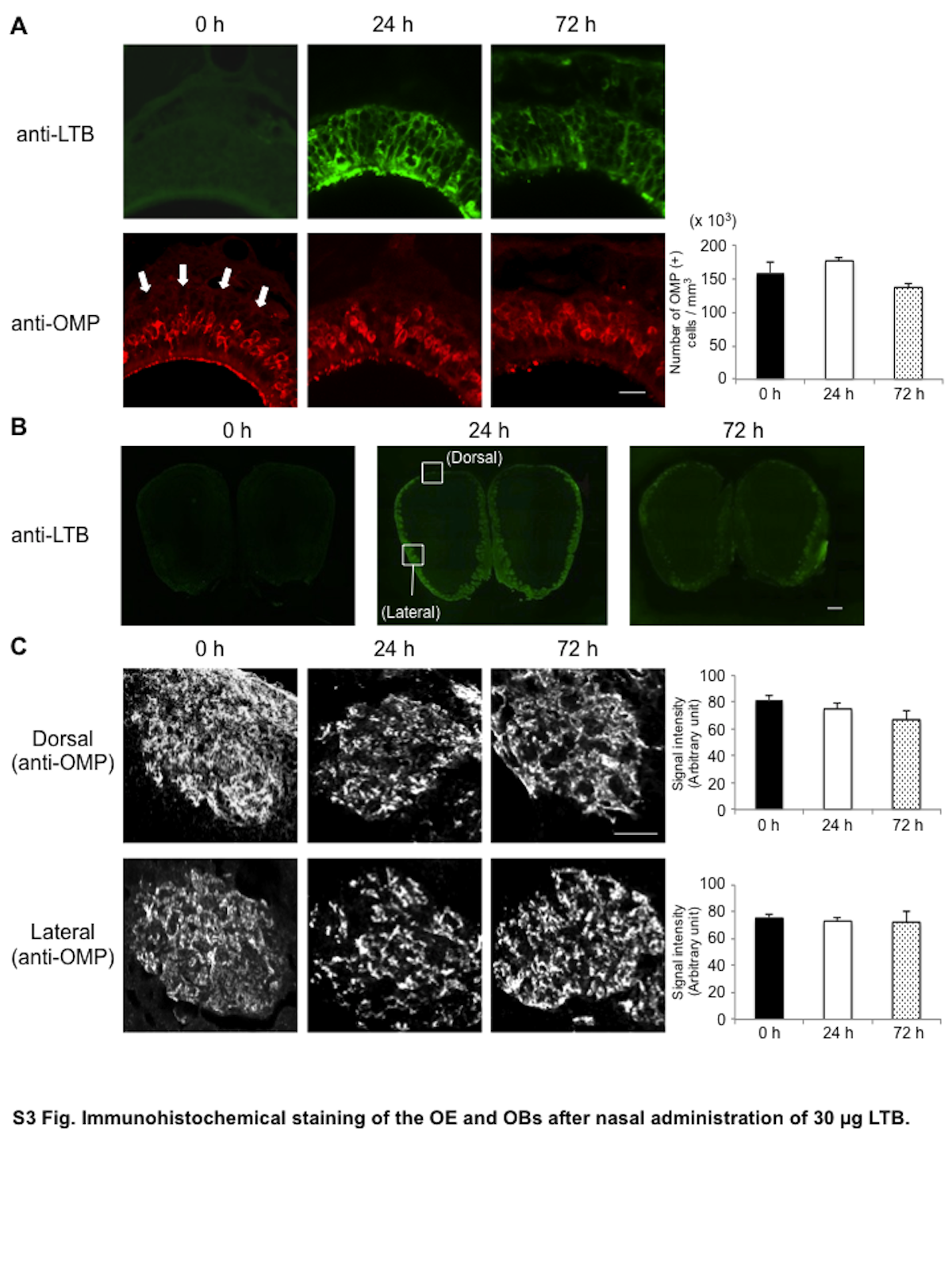

Supplement: S3 Fig — OE and OBs were obtained at 0 (untreated), 24, or 72 h after nasal administration of 30 μg LTB. (A) Frozen sections of OE were stained with an anti-LTB Ab (green) or anti-OMP Ab (red). White arrows indicate the olfactory nerve layer. The number of OMP-positive cells (mean ± 1 SD; n = 3 mice) in each experimental group is shown at the right. (B) Frozen sections of OBs were stained with an anti-LTB Ab. (C) Frozen sections of OBs were stained with anti-OMP Ab (converted to black and white images). Glomeruli at the dorsal and lateral surfaces of OBs (indicated by the boxes in panel B) are shown. The signal intensity (mean ± 1 SD; n = 3 mice) in each experimental group is shown. Data are representative of three independent experiments. Scale bars: (A, C) 20 μm, (B) 200 μm. (TIF) [file pone.0139368.s003.tif]

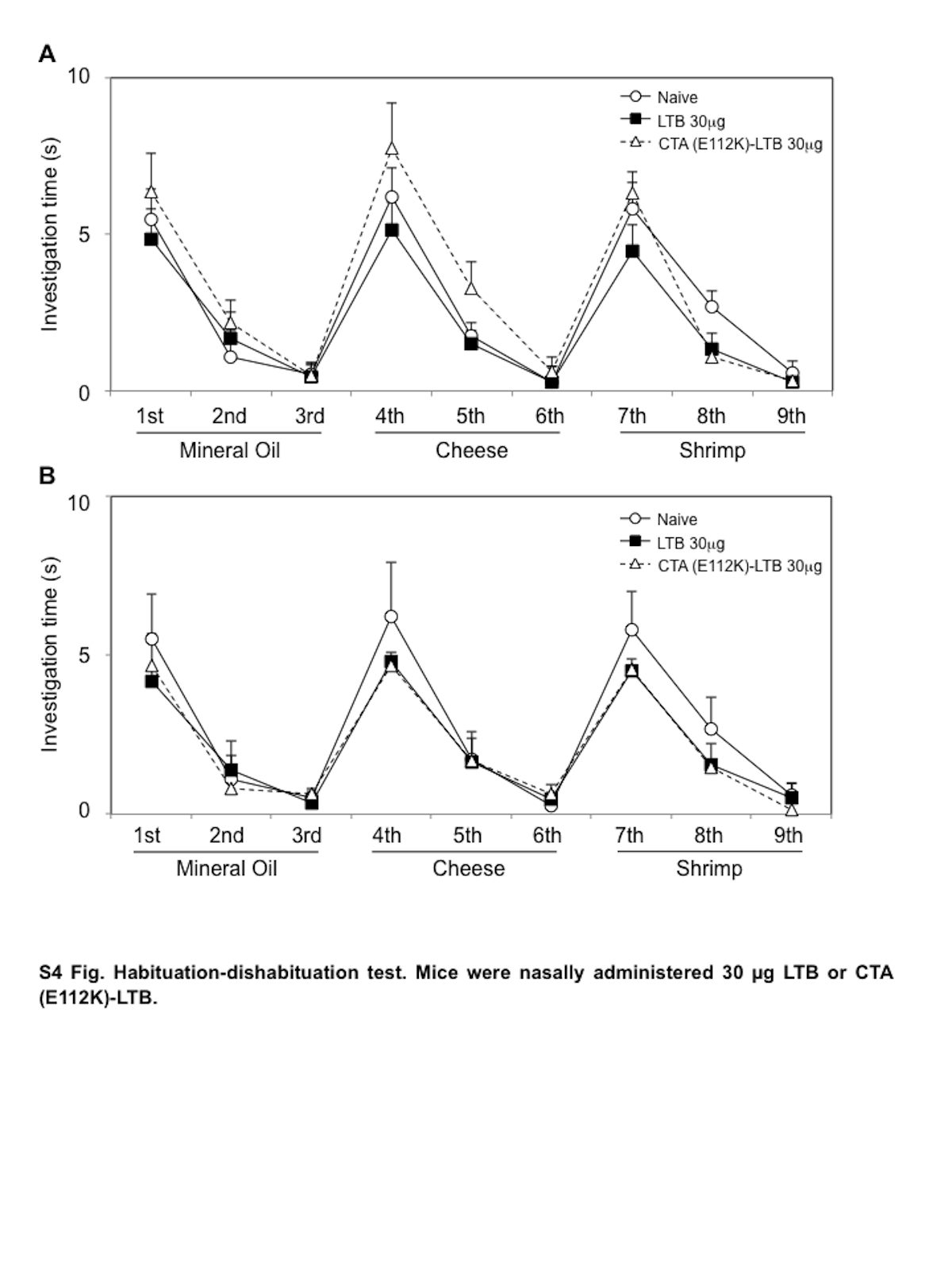

Supplement: S4 Fig — One day (24 h, A) or 3 days (72 h, B) after administration of the indicated drugs, the habituation—dishabituation test was performed with the indicated odors. Naïve (untreated) mice were used as controls. For each group, the time spent in investigation (mean ± 1 SD; n = 10 mice) in each experimental group during each 3-min period (with 15-min intervals) is shown. (TIF) [file pone.0139368.s004.tif]
